# The Role of Citicoline and Coenzyme Q10 in Retinal Pathology

**DOI:** 10.3390/ijms24065072

**Published:** 2023-03-07

**Authors:** Claudia García-López, Verónica García-López, José A. Matamoros, José A. Fernández-Albarral, Elena Salobrar-García, Rosa de Hoz, Inés López-Cuenca, Lidia Sánchez-Puebla, José M. Ramírez, Ana I. Ramírez, Juan J. Salazar

**Affiliations:** 1 Instituto de Investigaciones Oftalmológicas Ramón Castroviejo, Grupo UCM 920105, IdISSC, Universidad Complutense de Madrid, 28040 Madrid, Spain; 2 Facultad de Óptica y Optometría, Departamento de Inmunología, Oftalmología y ORL, Universidad Complutense de Madrid, 28037 Madrid, Spain; 3 Facultad de Medicina, Departamento de Inmunología, Oftalmología y ORL, Universidad Complutense de Madrid, 28040 Madrid, Spain

**Keywords:** citicoline, coenzyme Q10, CoQ10, eye, retina, glaucoma, non-arteritic ischemic optic neuropathy, age-related macular degeneration, diabetic retinopathy

## Abstract

Ocular neurodegenerative diseases such as glaucoma, diabetic retinopathy, and age-related macular degeneration are common retinal diseases responsible for most of the blindness causes in the working-age and elderly populations in developed countries. Many of the current treatments used in these pathologies fail to stop or slow the progression of the disease. Therefore, other types of treatments with neuroprotective characteristics may be necessary to allow a more satisfactory management of the disease. Citicoline and coenzyme Q10 are molecules that have neuroprotective, antioxidant, and anti-inflammatory properties, and their use could have a beneficial effect in ocular neurodegenerative pathologies. This review provides a compilation, mainly from the last 10 years, of the main studies that have been published on the use of these drugs in these neurodegenerative diseases of the retina, analyzing the usefulness of these drugs in these pathologies.

## 1. Introduction

Retinal diseases are one of the leading causes of visual impairment and blindness around the world, especially in developed countries [1,2]. Among them, neurodegenerative diseases such as glaucoma, age-related macular degeneration (AMD), and diabetic retinopathy (DR) are the most common, and their incidence is estimated to be increasing progressively [3,4,5].

The World Health Organization (WHO) in its 2019 world report on vision [6] states that “the aging of the population… coupled with changes in lifestyle (less time outdoors, increasing sedentary lifestyle, and unhealthy eating habits), are increasing the number of people with eye conditions and visual impairment”.

In these diseases, there is a neurodegenerative process that causes irreversible retinal damage leading to progressive vision loss [7]. Although there are treatments that attempt to slow the progression of these diseases, not all are equally effective. It is, therefore, necessary to develop new lines of research and alternative treatments or support for existing ones [7].

Citicoline and coenzyme Q10 are molecules that possess neuroprotective, antioxidant, and anti-inflammatory properties in the central nervous system. In fact, several studies have demonstrated the beneficial effects of these molecules in some retinal neurodegenerative diseases. Therefore, the aim of this work is to provide an exhaustive review of the studies published on this topic, mainly in the last ten years.

### 1.1. Citicoline

Citicoline (cytidine-5’-diphosphocholine or CDP-choline) is a mononucleotide composed of ribose, cytosine, pyrophosphate, and choline [8,9]. Citicoline is considered a drug with pleiotropic properties that activates the neuronal metabolism, stabilizes neuronal membranes, and promotes neurotransmission.

Citicoline is a precursor of both phosphatidylcholine, contributing to the synthesis of structural phospholipids in cell plasma membranes, and acetylcholine, an important neurotransmitter in cell metabolism [8,9,10].

The citicoline cycle is integrated into several metabolic pathways and its disruption can affect the distribution of various lipid metabolites [8]. If citicoline is taken orally, the compound is hydrolyzed to form choline and cytidine, which are used in the Kennedy cycle to generate phospholipids [9]; therefore, the administration of citicoline facilitates the exogenous supply of choline and cytidine [8].

Choline participates in numerous and significant neurochemical processes. It is a precursor and metabolite of acetylcholine [8], which is synthesized at the presynaptic ends of the axons of neurons [11]. Choline can be used from discrete reserves in the body and when released by the neurotransmitter acetylcholine [11].

Choline can be synthesized de novo from an endogenous pathway. However, the rate of synthesis by this pathway is not enough to satisfy the body’s choline demands; for this reason, choline is considered an essential nutrient in animal and human diets [11,12,13]. The average recommended choline intake for adult humans is approximately 550 mg/day for men and 425 mg/day for women, although the demand depends on factors such as age, sex, and weight [13,14,15].

For administration, citicoline can be found in the form of free base citicoline (dietary supplement) and sodium citicoline; the latter is mainly used as a drug for the treatment of neurological disorders. Citicoline is currently available as an intramuscular injection, oral formulation, and topical solution, to be instilled as eye drops [9,16]. Numerous experimental studies have been carried out administering citicoline in multiple conditions and pathologies [8,9]. In the eye, improvements in visual function have been demonstrated in patients with glaucoma, DR, non-arteritic ischemic optic neuropathy (NAION), and amblyopia [8].

### 1.2. Coenzyme Q10

Coenzyme Q10 (CoQ10) is a fat-soluble molecule whose molecular structure is similar to some vitamins. However, it is not a vitamin, since the cells of the human body can synthesize it de novo [17,18,19]. It is also called ubiquinone, given its ubiquitous presence in practically all cells of the human body [17,20].

This molecule is formed by a central ring of benzoquinone linked to a chain of 10 units of isoprenoid lipids [17,18,19].

The biosynthesis of CoQ10 involves three steps [18,19,21]:

(1) The synthesis of the benzoquinone structure, from tyrosine or phenylalanine. This forms the quinoid ring structure of the molecule;
(2) The synthesis of the polyisoprenoid side chain (made up of 10 isoprenoid units) from acetyl coenzyme A (CoA) through the mevalonate pathway;
(3) The union or condensation of these two structures to form CoQ10 [18,19].

CoQ10 exists in oxidized (ubiquinone) and reduced (ubiquinol) forms, and the normal function of CoQ10 involves a continuous interconversion between these two forms. In addition, there is a third, the partially reduced state (ubisemiquinone) [22].

CoQ10 is found in the inner mitochondrial membrane, in cell membranes, and in the blood, bound to both high-density and low-density lipoproteins [17,20]. It is involved in numerous biological processes, with the most important being the synthesis of adenosine triphosphate (ATP). CoQ10 participates in the electron transport chain that takes place during aerobic cellular respiration in the mitochondria, meaning it is essential for the production of energy in cells [17,20]. For this reason, CoQ10 is involved in the function of all cells, and especially those with high energy demands, which makes it essential for health [17].

The reduced form of CoQ10 (CoQ10H2) acts as a potent antioxidant. In fact, this molecule can counteract the effects of free radicals that can alter the lipids, proteins, and DNA of cells. This reduced form can also regenerate α-tocopherol (a form of vitamin E) with antioxidant action. In addition, CoQ10 can transport H+ in lysosomal membranes, allowing the proper functioning of lysosomes in degrading cellular debris [20].

Cells depend on biosynthesis to supply all the CoQ10 they need [17]. Thus, the endogenous CoQ10 levels are determined by both the rate of production and the rate of consumption in the body, and these levels can be altered in various pathologies [17,19]. Only a small amount of CoQ10 is obtained through the diet, and this is highly variable and depends on the type of diet.

There are three causes for CoQ10 deficiencies in the body: deficient synthesis, reduced intake, or an increase in its use by the cells. A deficiency in synthesis can be associated with an alteration of the genes involved in its synthesis, but also with an altered synthesis process [20].

The CoQ10 levels are fairly stable in cells, but the concentrations vary between different tissues and organs. In addition, the amount of CoQ10 decreases with age [21]. The possibility of increasing the CoQ10 levels in different organs or tissues through dietary supplementation has been widely explored in recent decades [21].

## 2. Citicoline and CoQ10: Relationship with Retinal Diseases

Once the general characteristics of both molecules have been described, the existing evidence that relates them to different retinal pathologies will be detailed below.

### 2.1. Treatments with Citicoline

#### 2.1.1. Citicoline and Glaucoma

Glaucoma is a chronic neurodegenerative pathology characterized by the loss of retinal ganglion cells (RGCs), leading to an irreversible loss of vision. The main risk factor is increased intraocular pressure (IOP). However, there are other pathogenic mechanisms involved in RGC cell death such as mechanical effects by an increased IOP, decreased neutrophine supply, hypoxia, oxidative stress, mitochondrial dysfunction, glutamate excitotoxicity, and neuroinflammation [23].

The neuronal membrane integrity is an important factor in the degeneration of axons and mitochondrial membranes in glaucoma [24]. These membranes are constituted by various phospholipids, such as phosphatidylcholine, acetylcholine, sphingomyelin, and cardiolipin, which can be derived from choline [24]. If the choline supplies are depleted or diminished, the hydrolysis of phosphatidylcholine and other phospholipids for obtaining this choline can occur, with consequent disruption of the cell membranes. Citicoline acts by providing choline, which prevents the disruption of the neuronal membrane and apoptosis in neurodegenerative processes, but it also produces acetylcholine [25]. These actions explain the neuroprotective properties of citicoline [26] mentioned in the studies discussed below.

Citicoline may also be involved in the synthesis of glutathione and glutathione reductase [8]. Glutathione is a metabolic product of choline and acts by reducing lipid peroxidation in the central nervous system, acting as an antioxidant [24]. Therefore, citicoline could also have an antioxidant role in neurodegenerative diseases induced by oxidative stress, such as glaucoma and ischemic neuropathies, among others [24].

Several studies have attempted to provide evidence that citicoline may be beneficial for glaucoma patients, suggesting that when administered in addition to ocular hypotensive therapy, it may produce beneficial effects [10]. The most recent studies that have analyzed the effect of citicoline administration on glaucomatous pathology in both experimental models and patients are presented below (Table 1).

The efficacy of citicoline in protecting the axons of the visual pathway has been demonstrated in an experimental model of glaucoma. This model involved the acute elevation of the IOP via anterior chamber perfusion with physiological saline in rats [27]. In this model, after the IOP elevation, different changes in the visual pathway were observed, such as alterations in myelin density and axon integrity and a decrease in anterograde axonal transport. However, the oral administration of citicoline (500 mg/kg for 3 weeks) significantly reduced the changes in the visual pathway without altering the IOP values, in addition to increasing the visual acuity (VA). These results were maintained three weeks after treatment interruption [27].

Other studies have analyzed the effects of citicoline administration in glaucoma patients using different routes of administration, such as eye-drop instillation or oral administration.

A series of studies analyzed the efficacy of citicoline administration in the form of drops in patients with open-angle glaucoma (OAG).

A randomized, double-blind study included a total of 78 patients with OAG and controlled IOP (18 mmHg) and had a duration of three years. [28]. In this study, patients were divided into two groups, control or placebo, and treated with instilled citicoline drops (formulated with 0.2 g of citicoline sodium salt). Several tests were performed, such as IOP measurements, visual field tests (VC) (Humphrey 10-2), and retinal-nerve-fiber-layer (RNFL) thickness tests (via optical coherence tomography (OCT)). After an analysis of the tests in both groups, statistically significant improvements in VF and RNFL thickness were found in the citicoline-treated group, supporting the neuroprotective role of this drug [28].

In another study, 47 eyes of patients with OAG and IOP controlled with beta-blockers (18 mmHg) were included [29]. Of these, 24 eyes were additionally treated with citicoline eye drops 3 times a day (2% citicoline sodium salt) for 4 months, followed by a 2-month washout period. An electrophysiological study was performed using a standard electroretinogram (PERG) and the visually evoked potential (VEP), finding in patients treated with citicoline a significant improvement in the electrophysiological response, which was maintained after the washout period [29].

Another study also confirmed the improved electrophysiological responses after the eye-drop administration of citicoline in patients with OAG [30]. In this study, a preliminary experimental phase was performed with mice, to test whether citicoline molecules at two different concentrations (1% and 2%) reached the vitreous and retina. The 2% citicoline solution was the one that showed systemic absorption and was subsequently used in a clinical phase with humans. The human study was a prospective, randomized, controlled, double-blind study that included 34 patients. The patients were administered a hypotensive treatment for 3 months, and 16 of them were additionally instilled with 2% citicoline 3 times a day for two months, followed by a one-month washout period. It was observed that the patients treated with topical citicoline showed significantly better electrophysiological responses (pERG and VEP) than untreated patients [30].

Other studies tested the efficacy of citicoline after oral administration in patients with OAG.

A first study tested the efficacy of oral citicoline associated with other substances (citicoline 500 mg, homotaurine 50 mg, and vitamin E 12 mg) in 109 OAG patients with controlled IOP (<18 mmHg) [31]. The study analyzed the IOP, VF, contrast sensitivity, and impact on glaucoma-related quality of life with the Glaucoma Quality of Life-15 (GQL-15) questionnaire. The study showed that the daily intake of citicoline–homotaurine–vitamin E in addition to a topical ocular-hypotensive treatment increased the total score of the contrast sensitivity test and the quality of life in patients with OAG.

The oral administration of citicoline alone also showed benefits for the visual system in glaucoma patients. In a study [32] conducted in 22 patients with OAG and controlled IOP (<21 mmHg), citicoline was administered orally at 600 mg/day (4 capsules of 150 mg) for at least 3 months. A functional study was performed at the initial visit and at 3 and 6 months using perimetry, PEV-pattern, and PEV-flash (P100 and P2 waves) measurements. In addition, a structural study was performed by measuring the thicknesses of the RNFL and the GCL using OCT. After the citicoline treatment, improvements in the VEP (increased amplitude and decreased latency) were observed. In addition, the OCT analysis showed that the RNFL thickness did not decrease during the study period.

Slightly lower doses of citicoline achieved beneficial effects in patients with glaucoma, as observed in a study [33] involving 60 OAG patients controlled with topical antihypertensive agents (IOP ≤ 18 mmHg). Thirty of them were given citicoline at a single daily dose of 500 mg in a 10 mL oral solution. White-on-white standard automated perimetry (SAP), RNFL- and GCL-thickness measurements with OCT were performed before starting treatment and at follow-up (6, 12, 18 and 24 months). The group treated with citicoline showed improvements in the tests performed with respect to the untreated group. Thus, the SAP values at 18 months were significantly higher in the citicoline-treated group and remained stable at all other times, while in the untreated group they decreased over time. Likewise, the RNFL and GCL thicknesses were significantly greater in the group treated with citicoline (at 12 months, remaining stable at the other times) with respect to the untreated group, in which there was a significant decrease.

Another study [34] verified the effects of citicoline (500 mg/day) on the VF values of 41 patients with OAG, following up with four VFs in 2 years, concluding that citicoline supplementation reduced the rate of glaucoma progression.

All of these studies demonstrate that both the instillation of eye drops and oral administration of citicoline produced improvements in visual function, indicating that these improvements were independent of the method of administration used.

**Table 1:** Summary of articles that relate citicoline to glaucoma, classified according to administration route.

Author	Study Population	Citicoline Dose	Results
**Topical instillation**			
Rossetti et al. [28]	Humans: OAG	0.2 g, 1 drop/day	↑ VF ↑ RNFL thickness
Parisi et al. [29]	Humans: OAG	2%, 3 drops/day	↑ pERG ↑ VEP
Roberti et al. [30]	Humans: OAG	2%, 3 drops/day	↑ pERG ↑ VEP
**Oral solution/tablet**			
van der Merwe et al. [27]	Rats: GM	500 mg/day	↑ VA ↓ Histological alterations of visual pathway
Marino et al. [31]	Humans: OAG	Neuprozin^®^: citicoline (500 mg), homotaurine (50 mg) and vitE (12 mg).	↑ CS ↑ GQL-15
Chitu et al. [32]	Humans: OAG	600 mg/day	↑ VEP
Lanza et al. [33]	Humans: OAG	500 mg/day	↑ SAP ↑ VF ↑ RNFL thickness
Ottobelli et al. [34]	Humans: OAG	500 mg/day	↑ VF

CS: contrast sensitivity; GM: glaucoma model; GQL-15: Glaucoma Quality of Life-15; OAG: open-angle glaucoma; RNFL: retinal nerve fiber layer; pERG: pattern electroretinogram; SAP: standard automated perimetry; VF: visual field; VEP: visual evoked potential; VA: visual acuity.

#### 2.1.2. Citicoline and Non-Arteritic Ischemic Optic Neuropathy

Arteritic ischemic optic neuropathy (AION) is the most common type of acute optic neuropathy in subjects over 50 years of age. It is caused by a deficit of blood supply to the optic nerve from the posterior ciliary arteries. It is characterized by sudden and severe vision loss, visual field deficit, peripapillary hemorrhages, and swelling of the optic nerve head. AION can be non-arteritic (NAION) or arteritic (AAION), with different prognoses. In NAION, partial resolution of the vision loss may occur, whereas in AAION there is a more detrimental deterioration of the vision, to blindness [35].

Parisi et al. studied the effects of citicoline oral solution (500 mg/day) administration for 6 months in a group of 36 patients with NAION. The VA, pERG, VEP, RNFL thickness, and Humphrey 24-2 VF results were analyzed, finding significant improvements in all parameters in the group treated with citicoline. These results were maintained after a 3-month washout period. This study confirmed the neuroprotective effect of citicoline in this pathology [36].

#### 2.1.3. Citicoline and Diabetic Retinopathy

DR represents one of the most important complications of diabetes and one of the main causes of visual impairment in people of working age in industrialized countries. Eighty-six percent of patients with type 1 diabetes mellitus (DM1) have DR. The risk factors for the development and progression of DR include the disease duration, poor metabolic control, hyperlipidemia, and hypertension. Although DR has classically been considered a microvascular disease, it is now known to have retinal neuronal involvement, such as the alteration of RGCs. The retinal neurodegenerative process could be caused by increased excitotoxic metabolites (e.g., glutamate), decreased folic acid and vitamin B12, or neuroinflammatory processes [10].

Retinal neuronal alterations can be recorded by alterations in CS tests, dark adaptation, the frequency-doubling-technology (FDT) perimeter, and multifocal electroretinograms (mfERGs). All of this is related to a cascade of different processes, such as the increased apoptosis of neuronal cells (RGCs and amacrine cells) or the impairment of glial cells, which are largely responsible for the integrity of the blood–retina barrier [37].

There are data supporting the role of citicoline in improving the function of RGCs and preventing their neurodegeneration. In the case of DR, citicoline has also been used topically as an eye drop to test its possible effects on this neurodegenerative disease (Table 2).

A study conducted in a diabetes model (db/db mice) analyzed the beneficial effect of citicoline on the retina [38]. The study was conducted using three groups of mice: a control group without diabetes, untreated diabetics, and diabetics treated with citicoline in liposomal solution (2% citicoline). Citicoline was administered topically twice a day for 15 days. A functional study was first performed, showing a decrease in ERG B-wave amplitude in diabetic mice. However, the diabetic animals treated with citicoline presented improvements in the amplitude of this wave. The citicoline administration also significantly prevented retinal degeneration caused by diabetes, by preventing glial activation and neuronal apoptosis when the study was performed using immunohistochemical and Western-blotting techniques [38].

The beneficial effect of citicoline has also been demonstrated in patients with diabetic retinopathy.

One study used a combination of topical citicoline (2%) with vitamin B12 in 20 patients with type 1 diabetes and mild signs of non-proliferative diabetic retinopathy (NPDR) for 3 years. This study showed decreases in microvascular damage and neuroretinal degeneration in the group treated with citicoline and vitamin B12 compared to the untreated group [39]. In addition, another study similar to the previous one, in which a functional study of the 21 patients with type 1 diabetes and mild signs of NPDR was performed, and showed an improvement in the macular electrophysiological response as measured by mfERG in diabetic patients treated with citicoline and vitamin B12 [40].

#### 2.1.4. Citicoline and Age-Related Macular Degeneration

AMD is the most common cause of blind registration in the developed world. Early AMD is characterized by drusen formation and pigmentary changes. Late AMD is characterized by geographic atrophy (dry AMD) and choroidal neovascularization (CNV, wet AMD). Wet AMD presents newly formed immature blood vessels growing from the choroid through Bruch’s membrane toward the outer retina. Wet AMD accounts only for about 10–15% of cases, but for 80–90% of resultant blindness cases. Anti-VEGF (vascular endothelial growth factor) therapies dramatically halt the progression of CNV in most wet-AMD patients, but there is no effective treatment for AMD patients with geographic atrophy. An important pathological feature of AMD is the accumulation of both focal (drusen) and diffuse-extracellular (basal) deposits in the macula, between the retinal pigment epithelium (RPE) and the adjacent Bruch’s membrane. These deposits lead to the dysfunction and subsequent death of the RPE and associated photoreceptors [41]. Oxidative damage is an important pathogenic mechanism in AMD, and substances with antioxidant characteristics may be useful in this pathology [42].

Currently, there is only one study that relates citicoline with AMD [43]. An in vitro analysis in citicoline-treated transmitochondrial AMD RPE cybrid cells showed that this drug (concentration of 1 mM) decreases apoptotic cell death, enhances cell viability, and reduces oxidative stress. The apoptotic cell reduction was studied using a flow-cytometry analysis, demonstrating decreases in the cell markers annexin V and propidium iodide in citicoline-treated AMD RPE cybrid cells. Additionally, the cells treated with citicoline showed significantly decreased levels of reactive oxygen species (ROS), which translated into oxidative-stress reductions. Finally, cybrid cells treated with citicoline present lower HIF-1α (hypoxia-inducible factor 1-alpha) and VEGF gene-expression rates than those in untreated cells; this is important, due to HIF-1α and VEGF activation being related to angiogenesis in choroidal neovascularization in AMD.

### 2.2. Treatments with CoQ10

#### 2.2.1. CoQ10 and Glaucoma

Elevated IOP affects the blood circulation of the eye, producing episodes of ischemia and reperfusion, leading to an oxidative-stress increase. This increase leads to mitochondrial dysfunction and also to the further generation of reactive oxygen spices (ROS) [7,22,31]. IOP elevation also leads to glutamate excitotoxicity (main excitatory neurotransmitter), which also causes mitochondrial dysfunction [29,44].

The loss of RGCs is associated with elevated RGC apoptosis [45,46,47]. Studies indicate that the main trigger for apoptosis is the opening of mitochondrial permeability transition pores (mPTP), followed by the segregation of apoptogenic molecules to the cytoplasm [46,48]. The mechanism by which CoQ10 appears to exert neuroprotection is due to its antioxidant activity and the stabilization of the cell membrane. It reduces the risk of mitochondrial depolarization and decreases the apoptosis induction. In addition, it also appears to inhibit glutamate excitotoxicity [25,49]. Both oxidative stress and glutamate excitotoxicity seem to contribute to the pathogenesis of glaucoma, so CoQ10 could be a therapeutic pathway for its treatment [22,45,46,50,51]. Moreover, a study [52] showed that CoQ10 prevented a significant loss of mitochondrial mass, suggesting an induction of mitochondrial biogenesis by this molecule.

CoQ10 is highly lipophilic, which represents a major limitation for its therapeutic use, as it is poorly absorbed and shows low bioavailability. For these reasons, an analog called idebenone has been developed [53]. The literature shows that it has neuroprotective properties against retinal degeneration [54], restoring mitochondrial function [22] and being capable of interacting with the mitochondrial respiratory chain and maintaining cellular energy production [55]. Some authors [56] have also found that idebenone also protected optic-nerve-head astrocytes from oxidative stress and apoptosis. Astrocytes are the main type of glial cells in the optic nerve head, and support the correct function of the ganglion-cell axons [22,47,52], also regulating the ionic balance and the metabolic and nutritional supply [56]. Another change that occurs in the glaucomatous retina is that the communication between glial cells and RGCs is damaged [47]. The loss of RGCs is accompanied by changes in the morphology of glial cells and in the expression of certain proteins, in a process called “glial activation”. Whether a cell survives or dies is determined by differences in the expression of some proteins, such as Bax and pBad, with some acting as promoters and others as inhibitors of apoptosis signals. Supplementation with CoQ10 has been shown to prevent glial activation by blocking oxidative stress, inhibiting Bax expression (pro-apoptotic), and increasing pBad (antiapoptotic) [44,50,57], resulting in the increased survival of RGCs [44,50].

Several routes have been used for the administration of CoQ10, such as eye drops for topical ocular instillation and oral- and intravitreal-administration routes. All of them have shown beneficial effects after administration in glaucomatous pathology (Table 3).

Studies involving the topical instillation of CoQ10 have observed that after application to the cornea, CoQ10 can reach the retina, but this depends on the dose and the time after administration [46]. In addition, CoQ10 is known to have poor aqueous solubility and low bioavailability, which increases when combined with α-tocopherol (a form of vitamin E), so most studies use this combination for the topical administration of CoQ10 [45].

It was shown that the topical application of CoQ10 (0.1%) solubilized with α-tocopherol polyethylene glycol succinate (TPGS) (0.5%) in micelles, produced a neuroprotective effect in a rat model of glaucoma [45]. Glaucoma was induced by hypotonic saline injected into two episcleral veins. The neuroprotective effect on the RGCs was achieved after the daily instillation of two 30 μL eye drops of CoQ10-TPGS. Moreover, the IOP remained unaffected, demonstrating that the neuroprotective role of CoQ10-TPGS was independent of the IOP.

In vitro studies performed on both primary-murine retinal mixed cultures (pMC) and the retinal neuronal cell line RGC5 pretreated with CoQ10-TPGS and exposed to cytotoxic insults (DMSO (dimethyl sulfoxide) or paraquat) demonstrated the neuroprotective effect of this substance [45].

Furthermore, the twice-daily topical instillation of CoQ10 + Vit.E (0.5% *w/v* TPGS (d-α-tocopheryl polyethylene glycol 1000 succinate) with 0.1% CoQ10 *w/v*) for 4 weeks produced a significant neuroprotective effect in a model of mechanical optic-nerve damage in Wistar rats. This effect was demonstrated by decreases in RGC loss, astrogliosis, and microgliosis (decreased expression of Iba-1 and GFAP) [58].

At this moment, there are no commercial eye drops available with CoQ10 only, but some are available in combination with vitamin E under the name COQUN^®^ (VISUfarma, Roma, Italy, coenzyme Q10 100 mg, d-polyethylene glycol 1000 succinate (Vitamin E TPGS)) [59]. There is also the COQUN^®^ modality as a food supplement with CoQ10 (COQUN^®^ OS) (VISUfarma, Madrid, Spain).

There are few studies that have analyzed the effect of CoQ10 administration in glaucoma patients.

To our knowledge, there is only one study analyzing the effect of COQUN^®^ in patients with OAG [59]. The study compared a group of OAG patients treated with topical β-blockers (GP) with another with the same hypotensive treatment plus COQUN^®^ (GC). After the visual-function testing, 60% of the patients in the GC group had improved retinal bioelectrical responses (increased pERG amplitudes and reduced implicit times). The patients who responded to treatment were those with greater retinal dysfunction at baseline. Additionally, 50% of the CG patients showed better VEP results, being the same as those who showed improved pERG results. The RTC parameter (an electrophysiological index) was also analyzed, and no significant differences were found between the two study groups. This study concluded that CoQ10 is not able to improve neuronal conduction along the posterior retinal visual pathways, but it does improve the visual cortical response [59].

The effect of CoQ10 in patients with pseudoexfoliative glaucoma has also been analyzed. A study was conducted in 64 patients and was a prospective, randomized clinical trial. The patients were treated with a topical instillation of coQ10 and vitamin E (COQUN^®^, 100 mg CQ10, 500 mg vitamin E TPGS) twice daily for one month. In patients treated with COQUN^®^, there was a decrease in oxidative stress, as they possessed a lower level of the enzyme superoxide dismutase [60].

Another form of CoQ10 administration is as a dietary supplement (Table 3). The positive effects of the oral administration of citicoline have been demonstrated in animal models of glaucoma.

The administration of CoQ10 (1.600–2.000 mg/kg body weight) in a mouse model of ischemia-reperfusion improved oxidative stress and prevented mitochondrial disruption following ischemic damage caused by increased IOP [44]. This suggests that the administration of CoQ10 as a dietary supplement could be a strategy to protect retinal neurons against ischemic damage.

Furthermore, in a genetic model of mice with glaucoma (DBA/2J mice), the administration of CoQ10 (1.600–2.000 mg/kg body weight) significantly prevented the damage produced by glaucomatous pathology by acting on several pathways, as follows [50]: (i) it promoted a 29% RGC survival rate, due to its action on mPTP in mitochondria, thereby preventing apoptosis; (ii) it blocked the apoptotic pathway by significantly decreasing the Bax-protein expression and increasing the pBad-protein expression; (iii) it partially preserved the RGC axons and decreased the GFAP expression in the glial lamina of the optic nerve head; (iv) it decreased glutamate excitotoxicity; (v) it improved oxidative stress by decreasing the protein-expression levels of NR1, NR2A, superoxide dismutase-2, and heme oxygenase-1; and (vi) it preserved the mtDNA content and Tfam (human mitochondrial transcription factor A)/oxo complex IV) protein expression, since the alteration of these parameters is related to mitochondrial disorders and early neurodegenerative changes related to retinal ischemia.

Ubiquinol, the reduced and active form of CoQ10, has also shown beneficial effects against glaucomatous damage in animal models when administered orally. The administration of 1% ubiquinol (1.600–2.000 mg/kg body weight) in the diet in an ischemia–reperfusion mouse model of glaucoma showed the increased survival of RGCs through the modulation of the apoptotic Bax/pBad S112/Bcl-xL pathway [57].

In another study [61] performed in a genetic model of glaucoma (DBA/2J mice), dietary supplementation with ubiquinol (1%) contributed to RGC survival through the modulation of mitochondrial biogenesis, the oxidative phosphorylation system (OXPHOS) complex, and Bax activation. In addition, it preserved the visual function against oxidative stress. Therefore, the authors concluded that ubiquinol could be used a therapeutic antioxidant to prevent mitochondrial dysfunction and oxidative stress in the glaucomatous degeneration of RGCs.

Finally, the third route of administration of CoQ10 that has been used is intravitreal (Table 3).

CoQ10 has been used in combination with other drugs such as anti-inflammatory substances to test its beneficial effect in glaucoma. In one study [62], a treatment involving the intravitreal injection of polylactic-co-glycolic acid (PLGA) microspheres (MSs) incorporating dexamethasone (D), melatonin (M), and CoQ10 was proposed, to create a sustained-release intraocular drug-delivery system. In the first part of the study, an in vitro model of glutamate-mediated neurotoxicity in R28 cells was used to evaluate the neuroprotective activity levels of different concentrations of the three active substances (D (50 μM, 100 μM, 200 μM), M (250 μM, 500 μM, 750 μM), CoQ10 (1 μM, 10 μM, 25 μM)) and vehicle controls. The treatment of cells with dexamethasone did not elicit a neuroprotective effect. Melatonin was neuroprotective at concentrations of 500 µM and 750 µM, whereas CoQ10 was neuroprotective at concentrations of 10 μM and 25 μM. To analyze the efficacy of the combination of these drugs in vivo, a rat model of ocular hypertension was used by injecting the episcleral veins with a hypersaline solution. Some animals received an intravitreal injection of DMQ-MSs microspheres, others received microspheres loaded with a single drug, and other animals were injected with empty microspheres. The DMQ-MSs-treated animals showed a significant neuroprotective effect on RGCs compared to the untreated ocular hypertension (OHT) controls. No such protective effect was observed in the groups treated with empty MSs and single-drug MSs. The authors suggested that multiply loaded PLGA MSs might constitute a novel therapeutic approach for the treatment of glaucoma [62].

**Table 3:** Summary of articles that relate CoQ10 to glaucoma, classified according to administration route.

Author	Study Population	CoQ10 Type/Dose	Results
**Topical instillation**			
Parisi et al. [59]	Humans: OAG	CoQ10 + Vitamin E; 2 drops/day	↑ pERG ↑ VEP
Ozotes et al. [60]	Humans: PG	CoQ10 + Vitamin E; 2 drops/day	↓ Oxidative stress
Acar et al. [58]	Rats: GM	CoQ10 + Vitamin E; 2 drops/day	↓ RGC loss ↓ Gliosis
Davis et al. [45]	Cell culture	20 μM CoQ10 with 57 μM TPGS (α-tocopherol)	↓ RGC loss
	Rats: GM	CoQ10 (0.1%) + TPGS (α-tocopherol) (0.5%)	↑ neuroprotection
**Oral supplementation**			
Lee et al. [44]	Mice: GM	CoQ10 (1.600–2.000 mg/kg)	↓ Oxidative stress and mitochondria alteration.
Lee et al. [50]	Mice: GM	CoQ10 (1.600–2.000 mg/kg)	↑ RGC survival ↓ Apoptosis ↓ Bax ↑pBad ↓ Oxidative stress ↓ Glutamate excitotoxicity
Ju et al. [57]	Mice: GM	CoQ10 (1.600–2.000 mg/kg)	↑ RGC survival
Edwards et al. [61]	Mice: GM	CoQ10 (1.600–2.000 mg/kg)	↑ RGC survival ↓ Oxidative stress
**Intravitreal injection**			
Arranz-Romera et al. [62]	Cell culture	CoQ10 (10–25 μM)	↑ neuroprotection
	Rats: GM	DMQ-MSs	↑ RGC survival

DMQ-MSs: dexamethasone, melatonin, and CoQ10 microspheres; CoQ10: Coenzyme Q10; GM: glaucoma model; OAG: open-angle glaucoma; PG: pseudoexfoliative glaucoma; RGC: retinal ganglion cell; pERG: pattern electroretinogram; VEP: visual evoked potential.

#### 2.2.2. CoQ10 and Age-Related Macular Degeneration

In AMD, RPE dysfunction plays a crucial role in its pathophysiology. Oxidative damage, increased apoptosis, and chronic inflammation seem key in the development of AMD, and some antioxidants such as CoQ10 (Table 4) are proposed as new therapeutic options [63]. Moreover, previous studies described the fact that most patients with AMD have lower levels of CoQ10 measured in plasma than the controls [64].

To investigate the effect of CoQ10 on AMD, RPE cell cultures, animal models, and patient studies have been used.

RPE cells (ARPE-19) were utilized to analyze the effect of CoQ10 or idebenone on hydrogen peroxide (H_2_O_2_) exposure, which decreases RPE cell viability and increases ROS formation. The treatment with idebenone significantly decreased the intracellular ROS formation. This demonstrates the potential effect of this molecule in reducing Bax and increasing Bcl-2, and consequently increasing cell survival [65].

Another study [48] showed that the topical instillation of CoQ10 inhibited the apoptosis generated by ultraviolet (UV) and γ radiation. To prove this, in vitro and in vivo experiments were performed. In vitro, the ARPE-19 cell line and RGC-5 rat cells were pre-treated with 10 μM of CoQ10 dissolved in 0.04% Lutrol F127, and then were irradiated with UV or γ rays. Additionally, the respiratory-chain blocker antimycin A (at 200 μM concentration) and fetal bovine serum restricted to 0.5% were also used as ischemia-mimetic apoptotic-stimulus non-inducers of free radicals. As a result, the CoQ10 increased the cell viability and lowered the retinal cell apoptosis in response to both the UV and γ radiation types and to chemical hypoxia or serum starvation.

To test the results in vivo, the study developed the animal model with UV irradiation using a high-pressure mercury arc lamp. During the three preoperative days, a combination of 0.2% CoQ10 in 10% Lutrol 127, 5% cremophor EL, 0,45% NaCl, and 0.001% benzalkonium chloride in H_2_O was applied 4 times per day as eye drops. CoQ10 protected all retinal layers from UV-induced apoptosis. The relationship between UV exposure and AMD is not yet very clear, but it is suggested that, due to the decrease in CoQ10 in the retina with age, improving its levels, especially in older patients, could have therapeutic value [48].

Intravitreal administration is a widely used form of drug delivery in AMD. It has the advantage of being an efficient way to deliver treatments to the posterior segment of the eye. However, it has the disadvantage of a high clearance rate of the drug, so repeated injections must be performed, resulting in ocular inflammation. In a novel study [54], a thermosensitive hydrogel including dexamethasone or ketorolac in combination with idebenone and D-α-tocopherol polyethylene glycol 1000 succinate (TPGS) was developed for intravitreal administration. With this hydrogel, micelles are formed, with the advantage that the drug release is sustained over time, thereby avoiding repeated injections. The combination of dexamethasone and idebenone promoted high oxidative protection in RPE-1 cells exposed to H_2_O_2_, with a viability rate of 86.2%. In addition, they decreased the Tumor necrosis factor (TNF)-α production significantly. Therefore, the authors proposed that these formulations could represent a new generation of neuroprotective treatments for retinal neurodegenerative diseases.

Several studies have been conducted in AMD patients using CoQ10 as a dietary supplement.

In the first study [66], 14 patients with early AMD were treated with a mixture of fatty acids (1320 mg/day), acetyl-L-carnitine (500 mg/day), CoQ10 (30 mg/day), and vitamin E (30 mg/day) for 3 months. The results showed a slight improvement in visual function after treatment. The controls were only treated with vitamin E, and worsened slightly. The same authors [67] conducted a similar study with a larger population group (106 patients). The study was randomized, double-blind, and placebo-controlled. The results of this study showed that the treatment group showed a decrease in the area covered by drusen and improvements in visual function, and only 2% of these worsened. However, in the placebo group, 17% worsened. These results suggest that an appropriate combination of the abovementioned compounds could stabilize the visual functions as well as improving ocular-fundus abnormalities in patients affected by early AMD [67].

**Table 4:** Summary of articles that relate CoQ10 to AMD.

Author	Study Population	CoQ10 Type/Dose	Results
Lulli et al. [48]	Cell culture	10 μM CoQ10	↑ viability of ARPE-19 and RGC-5 cell lines
	Rats: UV irradiation	0.2% CoQ10	↓ apoptosis
Feher et al. [66]	Humans: early AMD	Fatty acids (1320 mg/day) + acetyl-L-carnitine(500 mg/day) + CoQ10 (30 mg/day) + vitamin E (30 mg/day)	↑ visual function
Feher et al. [67]	Humans: early AMD	Fatty acids (1320 mg/day) + acetyl-L-carnitine(500 mg/day) + CoQ10 (30 mg/day) + vitamin E (30 mg/day	↑ visual function ↓ drusen area
Arend et al. [65]	Cell culture	Idebenone (1–100 μM)	↓Bax/↑ Bcl-2 ↑ viability ARPE-19 cells ↓ ROS
López-Cano et al. [54]	Cell culture	Dexamethasone (0.2%)+ ketorolac (0.5%) + idebenone (1 μM)	↓ Oxidative stress ↓ TNF-α

AMD: age related macular degeneration; CoQ10: Coenzyme Q10; ROS: reactive oxygen species; TNF-α: Tumor necrosis factor; UV: ultraviolet; RGC: retinal ganglion cell

#### 2.2.3. CoQ10 and Leber’s Hereditary Optic Neuropathy

Leber’s hereditary optic neuropathy (LHON) is caused by point mutations in mitochondrial DNA, resulting in mitochondrial dysfunction. RGCs are the main cells that are affected, as they are very sensitive to the disruption of ATP production and to oxidative stress. It is one of the most common optic neuropathies, and causes bilateral central-vision loss. Some patients also suffer from other neurological damage. Males are more affected than females. There is currently no curative treatment, and those that exist are supportive. Among the most promising treatments are gene therapies and the use of stem cells and CoQ10 analogs such as idebenone. These treatments attempt to restore ATP synthesis to neuroprotect RGCs [68].

Currently, idebenone (trade name Raxone^®^) is the first and only option approved in the European Union for the treatment of Leber’s hereditary optic neuropathy (LHON) [69] (Table 5).

Experimental studies have been conducted both in vitro and in vivo to study the usefulness of idebenone in LHON [55].

The in vitro studies used RGCs (RGC-5 cell line) and exposed them to rotenone, which is a mitochondrial complex I inhibitor. Another group of cells were exposed to rotenone plus idebenone. The results of this study showed that idebenone protected against the inhibition of mitochondrial complex I at concentrations greater than 10 nM. For the in vivo study, a mouse model of LHON was used, in which rotenone was injected intravitreally to provoke the pathology. The pre-treatment of these animals with idebenone protected against RGC loss, RNFL thinning, and gliosis. In addition, the animals recovered the loss of visual function, as measured by an optomotor test [55].

The evidence of the pharmacological activity and positive results obtained by idebenone in LHON in in vitro and in vivo studies led to the application of idebenone in humans.

A multicenter, double-blind, randomized, placebo-controlled trial was conducted in 85 LHON patients, in which the efficacy of idebenone (900 mg/day) was tested (RHODOS study). After treatment, there was an improvement in the mean visual acuity between the idebenone and placebo groups at 24 weeks. In addition, there was also a significant improvement in Tritan color contrast in the idebenone group at 12 weeks [70].

A subsequent study in a larger number of patients also demonstrated the benefit of idebenone for use in LHON. An open-label, multicenter, retrospective, non-controlled analysis of long-term visual acuity and safety in 111 LHON patients treated with idebenone (900 mg/day) was performed [69]. After treatment, 46% of the patients showed clinically relevant recovery of the best-corrected visual acuity (BCVA). Furthermore, an analysis of the treatment effect by treatment duration showed that the proportion of patients who recovered and the magnitude of recovery increased with the treatment duration. In responding patients, an average gain in BCVA of 0.72 logMAR (equivalent to more than seven lines on the Early Treatment Diabetic Retinopathy Study chart) was achieved. Among the patients with VA with less than 1.0 logMAR, 50% maintained that level of vision. This study also analyzed the efficacy, safety, and tolerability of the treatment, showing that ibedenone at a dose of 900 mg/day is a safe treatment option with almost no adverse effects [69].

Similarly, another multicenter, open-label, retrospective study analyzed the long-term outcome of idebenone (900 mg/day) treatment of Dutch patients with LHON on the visual function and RGC-layer thickness [71]. The results showed a clinically relevant recovery rate of 53% and a clinically relevant stabilization rate of 11%. The VA improvement in the clinically relevant recovery rate was 0.41 ± 1.54 logMAR, and was associated with the recovery of color discrimination. On the other hand, they also evaluated the thickness of the ganglion cell complex (GCC) and retinal nerve fiber layer (RNFL) by OCT; however, even with treatment there was an irreversible loss of thickness in these layers. This was in contrast to the observations in the mouse model of LHON previously mentioned [55].

The results of these studies gave sufficient evidence that the idebedone treatment improved vision in patients with LHON. Therefore, given the rarity and severity of the disease [47,72,73], idebedone was approved in 2015 by the European Medicines Agency to treat LHON.

In 2017, an international consensus statement [74] recommended the use of idebenone during the first year after disease onset. It is important that treatment with idebenone be started as early as possible and continued for more than 2, years to maximize its efficacy. The main problem in LHON is that the diagnosis is usually made late, after the first symptoms of visual loss, and may take weeks or months. In addition, it is usually initiated when the second eye starts to be affected [69].

Both the RHODOS study [70] and the two studies mentioned above [69,71] included patients with LHON in whom treatment with idebedone was initiated in the first 2 years after disease onset. However, there are limited data on what occurs when this treatment is initiated more than 5 years after the disease onset. A study [73] was performed in patients with chronic LHON in whom treatment with idebedone was started later than 5 years and up to 51 years after disease onset. This study demonstrated for the first time an improvement in visual function in chronic LHON patients with late-onset treatment with idebedone. All patients showed an improvement in VA of two to three logMAR lines. Specifically, one eye improved from blindness to severe visual impairment, four eyes improved from severe to moderate impairment, and three eyes from moderate to mild vision loss. Improvements in the visual field were also observed. These improvements appeared to be the result of the reactivation of signal transduction in dysfunctional but still surviving RGCs. This study had few patients, but the results indicated a beneficial effect of idebedone on the visual function in LHON patients with established optic atrophy [73].

**Table 5:** Summary of articles that relate CoQ10 to LHON.

Author	Study Population	CoQ10 Type/Dose	Results
Heitz et al. [55]	Cell culture Mouse: LM	Idebenone (1–100 μM)	Cytoprotective ↓ RGC death ↓ Gliosis
Klopstoc et al. [70]	Humans (LHON)	Raxone (900 mg/day)	↑ VA ↑ Tritan color contrast
Catarino et al. [69]	Humans (LHON) Retrospective	Raxone (900 mg/day)	CRR 46% ↑ VA
Everdingen et al. [71]	Humans (LHON) Retrospective	Raxone (900 mg/day)	CRR 53% CRS 11% ↑ VA
Pemp et al. [73]	Humans LHON chronic	Raxone (900 mg/day)	↑ VA

CRR: clinically relevant recovery; CRS: clinically relevant stabilization; LM: Leber’s-hereditary-optic-neuropathy model; LHON: Leber’s hereditary optic neuropathy; RGC: retinal ganglion cell; VA: visual acuity.

#### 2.2.4. CoQ10 and Retinitis Pigmentosa (Table 6)

Retinitis pigmentosa (RP) is a heterogeneous group of diseases in which the progressive degeneration of the photoreceptors occurs. This causes an early clinical feature called night blindness that is followed by a loss of peripheral vision, finally affecting the central vision. Another clinical feature is the bone-spicule pigment deposits in the fundus of the patient’s eye. This is a pathology that affects 1/4000 people worldwide [22]. RP is thought to be caused by a mutation of one or more genes. Recent studies have revealed that mitochondrial dysfunction and oxidative damage play a critical role in photoreceptors and in the pathogenesis of RP [22,75]. Until now, more than 100 genes have been associated with RP; although most of them are expressed in photoreceptors or in the RPE, there are many that directly or indirectly affect mitochondrial functions [75].

**Table 6:** A summary of articles that relate CoQ10 to RP and DR.

Author	Study Population	Coq10 Type/Dose	Results
**Retinitis pigmentosa**			
Fernandez-Sanchez et al. [76]	Mouse: RPM	CoQ10	↑ ERG ↑ Photoreceptor preservation
Lodi R. et al. [77]	Human: RP	CoQ10 (100 mg/day)	↑ Brain energy reserve
**Diabetic retinopathy**			
Daniel et al. [78]	Rat: DRM	Idebenone (0.03 M) Elamipretide (0.03 M) SCQs (0.01 M #37, 0.02 M #77) 1 drop day	↑ VA ↓ Oxidative damage ↓ RGC loss, ↓ Reactive gliosis ↓ Vascular leakage ↓ Retinal thinning
Rodríguez Carrizalez et al. [79]	Human: NPDR	CoQ10 (400 mg/day)	↓ Oxidative stress

CoQ10: Coenzyme Q10; VA: visual acuity; DRM: diabetic retinopathy model; ERG: electroretinogram; NPDR: non-proliferative diabetic retinopathy; RGC: retinal ganglion cell; RPM: Retinitis pigmentosa model; RP: Retinitis pigmentosa; SCQs: short-chain quinones.

The combination of different antioxidants (α-tocopherol, ascorbic acid, 4-benzoic acid, porphyrin, and α-lipoic acid) has been shown to slow or reduce cone death in different animal models of RP. Since CoQ10 has antioxidant capacity, its usefulness in RP has been investigated [22].

In an experimental model of RP (Rd10 mice), the protective effect of CoQ10 was analyzed [76]. Two groups of mice were used, whereby the pups of one group were fed a diet supplemented with CoQ10 from p14 to p25, while the control group was fed a normal diet. The retinas were evaluated with ERG, OCT, and immunohistochemical techniques. In CoQ10-treated animals the following results were found: (i) higher a-waves in the scotopic light-induced retinal response; (ii) a higher density of synaptic contacts between photoreceptors and bipolar neurons, and of the dendritic tips of horizontal cells; and (iii) the better preservation of cone- and rod-axon terminals. Therefore, the authors concluded that CoQ10 was able to delay the loss of retinal function and the disruption of synaptic contact in Rd10 mice, indicating that this molecule could be of use in RP.

Studies on CoQ10 and RP in humans are scarce. An old study conducted in three RP patients with oral administration of CoQ10 at a dose of 100 mg/day for 2.5 months showed via phosphorous magnetic resonance spectroscopy (allowing the analysis of the bioenergetics of human tissues) that the energy reserve in the brain was increased in two of the three patients in the study, indicating that CoQ10 could improve the mitochondrial function in the brains of RP patients [77].

#### 2.2.5. CoQ10 and Diabetic Retinopathy (Table 6)

Oxidative stress and mitochondrial dysfunction have also been linked to DR. Hyperglycemia induces the overproduction of ROS by mitochondria. Therefore, substances that decrease oxidative stress, either by scavenging ROS or by enhancing antioxidant expression, could have a therapeutic role in DR [22,78].

In a study performed in a rat model with type 2 diabetes, the beneficial effect of mitoprotective compounds including idebenone (CoQ10 analogue) was analyzed [78]. Apart from idebenone (0.03 M), elamipretide (0.03 M) and short-chain quinones (SCQs) (0.01 M #37, 0.02 M #77) were used. When a 65% loss rate in visual function was detected in these animals, these drugs were instilled once daily in one eye, leaving the contralateral eye as a control. After 3 weeks of treatment, the vision was significantly restored, from 35% to 58–80%, in the treated eyes. However, the vision continued deteriorating in the untreated eyes. The drugs that provided the best results were the SCQs. These mitoprotective drugs also protected against oxidative damage, loss of RGCs, reactive gliosis, vascular leakage, and retinal thinning. Therefore, the authors concluded that mitoprotective compounds, and especially SCQs, had great potential as drug candidates for the treatment of DR [78].

Another study performed in patients with non-proliferative DR [79] evaluated the effect of CoQ10 and combined antioxidant therapy (CAT) on markers of oxidative stress in this pathology. The first group was supplemented with CoQ10 (400 mg), the second group received CAT (10 mg of lutein, 4 mg of astaxanthin, 1 mg of zeaxanthin, 180 mg of vitamin C, 30 mg of vitamin E, 20 mg of zinc, and 1 mg of copper), and the third group received a placebo. In the CoQ10 and CAT groups, the levels of oxidative and antioxidant biomarkers improved significantly.

## 3. Citicoline and CoQ10: Concluding Remarks

Citicoline forms parts of cell membranes and is essential in neurotransmission processes [8]. CoQ10 can carry out the functions of the mitochondria, and is vital in terms of energy production [17,18,19,20] and for controlling ROS levels [14]. Furthermore, CoQ10 has been shown to be one of the most effective antioxidants available [17,21,22]. As common characteristics, both molecules can be synthesized de novo by the organism [12,17,18,19], although in citicoline there is an almost absolute dependence on exogenous diet sources [12,13].

In addition to the retinal pathologies described in the literature review, the use of these molecules in other ocular diseases and conditions is noteworthy. Citicoline has been used in amblyopia [80,81], myopia [82], and restoring corneal sensitivity after laser-assisted in situ keratomileusis (LASIK) [83]. Additionally, CoQ10 has been used in spaceflight-associated neuro-ocular syndrome [84], in the prevention of keratocyte apoptosis after photorefractive keratectomy (PRK) [85,86], and even in diseases that have a hereditary component such as those already described (RP and LHON).

According to the evidence collected in this bibliographical review, it seems that the administration of citicoline and CoQ10 could have a promising effect in controlling the progression and benefiting the visual function of many retinal pathologies such as AMD, DR, and glaucoma. However, in the case of CoQ10, most of the studies have been in vitro or in animal models. Of the studies conducted in humans, the study carried out with idebenone in patients with LHON stands out. This disease is relatively rare, resulting in a very severe visual impairment, and to date is without treatment, so these special circumstances led the European Union to approve CoQ10 therapy in these patients [47,72,73]. However, although the results are generally positive, there is some controversy that makes it necessary to continue research in this area. In a recent study [53], in agreement with previous research by other authors [87,88,89], it was explained that idebenone could also have harmful effects, such as the opening of mitochondrial permeability transition pore (mPTP) [88] or the inhibition of the chloride channel activated by calcium [89]. In addition, recent evidence has suggested that there is a big difference between these two compounds (idebenone and CoQ10); idebenone has a lower molecular weight and the ability to cross membranes, it cannot activate complex I, and it can be reduced by NQO1 (NAD(P)H Quinone Dehydrogenase 1) [53]. Thus, the pharmacokinetics, bioactivation, and modulation of cellular energy production differ in both molecules [90]. Moreover, idebenone could change from an antioxidant to pro-oxidant, depending on its concentration and the expression of the enzyme NQO1, and this could generate ROS; it seems that if the cells have little NQO1 they will be more vulnerable to idebenone. Taken together, this may explain why some patients respond and others do not to treatment with idebenone [53]. Therefore, the literature [90] reports that idebenone should not be referred to as an “analog” of CoQ10.

Glaucoma is the pathology for which the most evidence is found for both citicoline and CoQ10. There have been numerous studies in which citicoline has been administered in humans with beneficial results, while in the case of CoQ10 there is less evidence. However, the study in which COQUN^®^ eye drops were used is noteworthy [59]. A new randomized, double-blind, placebo-controlled clinical trial is currently being carried out with COQUN^®^ [91,92], the results of which will be interesting to consider.

The mechanism of action of citicoline in glaucoma is multifactorial, including, among others, the preservation of cardiolipin and sphingomyelin; the restoration of phosphatidylcholine; the stimulation of glutathione synthesis; the prevention of neural apoptosis; and the synthesis of glutathione, myelin, and acetylcholine [28]. In the studies analyzed in this bibliographic review, improvements in VA [27], VF [28,33,34], electrophysiological tests [29,30,32], measurements of RNFL thickness [28,32,33], CS [31] or subjective character tests such as the GQL-15 test were found [31].

The mechanisms through which CoQ10 exerts retinal neuroprotection in glaucoma are due to its antioxidant activity, its ability to stabilize the cell and mitochondrial membrane, and the inhibition of excitotoxicity by glutamate [22,45,46,49,50]. CoQ10 has also been shown to have the ability to inhibit or stimulate the expression of pro-apoptotic or antiapoptotic proteins. Several studies have confirmed the inhibition of RGC apoptosis and glial activation after the administration of CoQ10 [44,50,61]. A study also concluded that it improves the visual function by measuring VEPs and by means of an optomotor system with the use of CoQ10 [61]. In this line, another study concluded that electrophysiological responses are improved, but not in all cases [59]. It should be noted that in the first study they used oral supplementation and in the second they used a topical instillation of COQUN^®^. Thus, there is great heterogeneity in the design of the studies, which makes it difficult to compare their results.

In DR, the neurodegenerative process mainly affects the RGCs and amacrine cells, which consequently produce structural thinning of the neuronal and axonal layers of the retina in the macular area [10]. The topical instillation of citicoline at 2% has been shown to have a protective effect against retinal neurodegeneration [38], as well as causing improvements in the macular electrophysiological response [39]. Oxidative stress, mitochondrial dysfunction, and ROS overproduction have been linked to DR, and it is, therefore, thought that CoQ10 may have a neuroprotective effect in this disease. The instillation of idebenone in eye drops improved the VA in an animal model of type II diabetes [78]. CoQ10 has also been shown to improve oxidative and antioxidant biomarkers in non-proliferative DR [79].

In AMD, the use of CoQ10 in combination with other compounds seems to reduce the progression of retinal damage [66,67] and even reverse part of the accumulation of drusen [67]. In in vitro studies, it seems that both idebenone and CoQ10 exert a protective effect on RPE [56,84].

It is remarkable that citicoline and CoQ10 affect many neuroretinal pathologies, as they are very different to each other. The variety of mechanisms of action of citicoline could result in an improvement in the integrity of the neuronal membrane, reducing oxidative stress and improving the synthesis of neurotransmitters such as acetylcholine. These applications could explain the multiple neurotherapeutic effects of citicoline in glaucoma and in neurodegenerative diseases in general [28]. After analyzing the literature, it can be concluded that in neuroretinal pathologies there is oxidative stress, ROS accumulation, and mitochondrial dysfunction. Since CoQ10 has effects on oxidative stress and mitochondrial integrity, there is evidence that the use of this compound in these retinal pathologies may have great potential.

Some authors propose the combined use of different molecules as treatments or adjuvant therapies in some diseases [63], but no studies have been found that combine citicoline and CoQ10. However, COQUN Combo^®^ is commercialized as a food supplement, consisting of 250 mg of citicoline and 100 mg of CoQ10 (VISUfarma, España). According to the laboratory, it is recommended that people take 2 COQUN Combo^®^ tablets daily, which would mean a total dose of 500 mg of citicoline and 200 mg of CoQ10. In this review, it was pointed out that the recommended dose of citicoline in adults is between 425 and 550 mg/day [7], although these amounts have not been updated since 1998 [93];more specifically, in the reviewed studies, the doses of citicoline used orally equaled 500 mg/day [31,33], and only one study used a higher amount, of 600 mg [32]. In the case of CoQ10, there is less dependence on the diet, but the evidence indicates that biosynthesis decreases with age, and supplementation could be beneficial to improve CoQ10 levels in the body.

Given that supplementation with citicoline and CoQ10 has few or no side effects, it could be feasible to carry out studies in humans that compare control groups with others supplemented with a combination of both molecules, such as COQUN Combo^®^, to assess whether they produce clinically significant effects on the progression of different neuroretinal pathologies or on parameters that evaluate visual function. In summary, more human studies would be needed to corroborate whether citicoline and CoQ10 in dietary-supplement form have clinically significant effects, so that health professionals can recommend them based on solid, substantiated evidence. On the other hand, it would be useful for health professionals, who at some point may have contact with patients affected by this type of neurodegenerative disease, to publish clinical guidelines that specify how and in what cases to recommend citicoline and CoQ10 food supplements, because although they are not patentable drugs, but rather nutraceuticals, there should be a consensus on their uses and applications.

## 4. Conclusions

**Figure 1:**
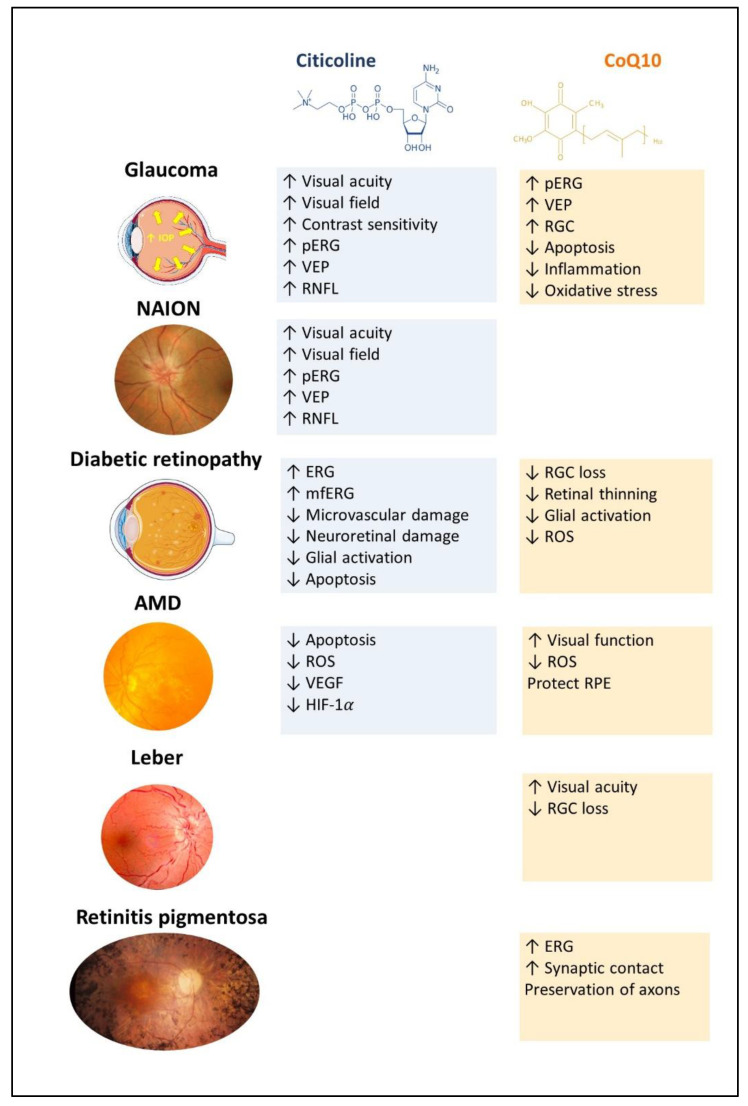
Schematic summary of the main ocular benefits of citicoline and CoQ10 in different ocular pathologies (created in part with smart.servier.com). ERG: electroretinogram; mfERG: multifocal electroretinogram; pERG: pattern electroretinogram; RGC: retinal ganglion cell; ROS: reactive oxygen spices; RNFL: retinal nerve fiber layer; VEGF: vascular endothelial growth factor ; VEP: visual evoked potential.

Both citicoline and CoQ10 are essential compounds for the proper functioning of the body in general and the visual system in particular. The literature review shows that these molecules could serve as treatments or adjuvant therapies for different retinal pathologies, due to their very diverse mechanisms (Figure 1), highlighting, in the case of citicoline, the activation of the neuronal metabolism and stabilization of neuronal membranes In addition, CoQ10 has antioxidant properties, stabilizes mitochondrial membranes, inhibits and stimulates pro- and antiapoptotic proteins, respectively, and inhibits glutamate excitotoxicity. Studies linking both molecules in the treatment of many retinal pathologies have shown promising results but can only be considered a first step towards their use in clinical practice for patient benefit.

## Figures and Tables

**Table 2:** Summary of articles that relate citicoline to DR, classified according to administration route.

Author	Study Population	Citicoline Dose	Results
**Topical instillation**			
Bogdanov et al. [38]	Mice: DM	2%, 2 drops/day	↑ ERG
			Prevent glial activation and apoptosis
Parravano et al. [39]	Humans: NPDR	2% (+VitB12),	↓ Microvascular damage
		3 drops/day	↓ Neuroretinal degeneration
Parisi et al. [40]	Humans: NPDR	2% (+VitB12) 3 drops/day	↑ mfERG

DM: diabetes model; NPDR: non-proliferative diabetic retinopathy; ERG: electroretinogram; mfERG: multifocal electroretinogram.

## Data Availability

Not applicable.

## Abbreviations

ALA	α-lipoic acid
ARMD	age-related macular degeneration
ATP	adenosine triphosphate
CAT	combined antioxidant therapy
CRR	clinically relevant recovery
CRS	clinically relevant stabilization
CoQ10	coenzyme Q10
D	dexamethasone
DMSO	dimethylsulfoxid
DR	diabetic retinopathy
ERG	electroretinogram
ETDRS	Early Treatment Diabetic Retinopathy Studio
FDT	dual-frequency perimetry
GCL	ganglion cell layer
GQL-15	Glaucoma Quality of Life-15
HO-1	heme oxygenase-1
LASIK	keratomileusis, laser-assisted
LHON	Leber’s hereditary optic neuropathy
M	melatonin
MSs	microspheres
mfERG	multifocal electroretinogram
mmHg	millimeters of mercury
MNs	mitochondrial nutrients
mPTP	mitochondria permeability transition pores
NPRD	nonproliferative diabetic retinopathy
IOP	intraocular pressure
OAG	open-angle glaucoma
OCT	optical coherence tomography
PEMT	phosphatidylethanolamine methyltransferase
pERG	patterned electroretinogram
PLGA	polylactic-co-glycolic acid
PRK	photorefractive keratectomy
RNFL	retinal nerve fiber layer thickness
RGCs	retinal ganglion cells
ROS	reactive oxygen species
RP	retinitis pigmentosa
RPE	retinal pigment epithelium
SAM	S-adenosylmethionine
SAP	standard white-on-white perimetry
SCQs	short-chain quinones
SOD2	superoxide dismutase 2
TPGS	α-tocopherol polyethylene glycol succinate
VA	visual acuity
VEP	visually evoked potential
WHO	World Health Organization
